# Observations on Pulmonary Tuberculosis in Infancy and Early Childhood

**Published:** 1907-09-21

**Authors:** William P. S. Branson

**Affiliations:** Assistant Physician, East London Hospital for Children


### OBSERVATIONS ON PULMONARY TUBERCULOSIS IN INFANCY AND EARLY CHILDHOOD.

By WILLIAM P. S. BRANSON, M.D., M.R.C.P., Assistant Physician, East London Hospital for Children.

In the lungs, as elsewhere, tuberculosis presents during early childhood a tendency to rapid caseation and an easy diffusion which contrast strongly with the relative chronicity of the common adult type of the disease, and the younger the victim the more marked is this tendency. Thus, during the first two years of life the usual varieties of pulmonary tuberculosis are (1) a generalised miliary infection, and (2) a sub-acute caseating broncho-pneumonia. Anything approaching the anatomical picture of chronic phthisis is a notable rarity during infancy. In the first place, the singular constancy with which the disease selects the apices of the lungs in maturer life has no counterpart in early childhood. At this epoch we find that, although the upper lobe still suffers most, it is the middle zone of the lung that presents the most marked lesions, the infection spreading apparently from the root by way of the smaller bronchial and intra-pulmonary lymphatic glands. In the second place evidence of obsolescence of a tuberculous process, which in later life is a commonplace of the dead-house, is quite rare in infancy. For the rest one may say that with each added year of life the type of the disease shows a slow but constant change towards the chronicity which characterises pulmonary tuberculosis in the adult, until at or about the end of the first lustrum the metamorphosis is to all intents and purposes complete. At all periods of childhood one meets occasionally with the acute pneumonic variety of tuberculosis, marked by massive caseous consolidation of a whole lobe, or more often of the whole of a lung, and ending, if life be sufficiently prolonged, in extreme caseous disintegration of the affected viscus. Lastly, of all tuberculous lesions in childhood caseation of the bronchial lymphatic glands is the commonest. Such caseation is constantly present when the lungs are tuberculous, and often when they are not.

It is usual to say that the predisposing causes of pulmonary tuberculosis in childhood are heredity, lack of ventilation, measles, and whooping-cough. This is not the whole truth. An investigation * showed me that of 135 children dying of tuberculosis at the East London Hospital, 25 per cent. only came of tuberculous stock, while no less than 50 per cent. had never had either measles or whooping-cough. This feature becomes still more striking when we consider tuberculous deaths before the end of the second year alone, for of such children no fewer than 70 per cent. were free from a history of the two zymotic diseases mentioned. It is clear, therefore, that none of these three causes, admittedly predisposing as they are, is specific in its action, and probable that the two last operate through the catarrhal lesions of mucous membranes which are their common associates; and since it is certain that there is no more potent factor in the production of catarrhal lesions than deficient ventilation and unhygienic surroundings, we may legitimately conclude that measles and whooping-cough are only accidentally to blame.

* *Brit. Med. Jour.*, Jan. 14, 1905, p. 72.

The identification of pulmonary tuberculosis during the first three or four years of life meets a peculiar difficulty in the fact that no sputa are available for microscopic examination, since young subjects habitually swallow their expectoration. In the absence of such a guide the distinction between a tuberculous and a simple broncho-pneumonia provides a problem always perplexing and not infrequently for the time being insoluble. It is occasionally possible to detect large, hard glands in the episternal notch or above the clavicles, an occurrence which is, in my opinion, diagnostic of pulmonary tuberculosis. But apart from this, or some evidence elsewhere that the child is the host of the tubercle bacillus, judgment must be suspended. Dr. Eustace Smith teaches that a diagnosis of pulmonary tuberculosis is unwarranted and unwise unless the clinical characters and physical signs of the disease can be reconciled on no other supposition. There can be no question, I think, of the justice of this aphorism. Simple broncho-pneumonia, especially when complicating whooping-cough, may simulate the tuberculous disease in every particular. One of the commonest errors of out-patient practice is the condemnation as tuberculous of a broncho-pneumonia whose simple character is either proved to demonstration, or at least strongly corroborated, by subsequent events.

Acute miliary tuberculosis of the lung is commonly an incident of a general infection with the tubercle bacillus. It presents as a rule no physical signs. For the existence of continued respiratory embarrassment without physical evidence of a lung lesion permits of no other interpretation than miliary tuberculosis of the lung. It is true that occasionally the physical signs of lobar pneumonia may delay their appearance for some days, but in such a case the urgency of the symptoms is more marked and the fever of a higher grade than is the rule with miliary tuberculosis of the lung.

Massive tuberculosis of the lung may for a time give rise to great doubt as to the diagnosis, and is most likely to be confounded with empyema. The onset is more or less abrupt. There is high fever, and the whole of one lobe, or the whole of one lung, gives physical evidences pointing to consolidation or effusion. Within a few days the fever begins to intermit and assumes a hectic character. The suspicion of a purulent pleurisy naturally arises, and when an exploratory puncture yields pus, as is likely to be the case, a diagnosis of empyema is an easy blunder. The pus in such cases is supplied by the liquefaction of the caseous lung. I have twice seen these caseous pulmonary abscesses opened under the belief that they were pleural. Even bacteriological examination may be misleading unless the tubercle bacillus be specifically sought for.

